# An intrinsic timer specifies distal structures of the vertebrate limb

**DOI:** 10.1038/ncomms9108

**Published:** 2015-09-18

**Authors:** Patricia Saiz-Lopez, Kavitha Chinnaiya, Victor M. Campa, Irene Delgado, Maria A. Ros, Matthew Towers

**Affiliations:** 1 Instituto de Biomedicina y Biotecnología de Cantabria, IBBTEC (CSIC-Universidad de Cantabria), Santander 39011, Spain; 2 Bateson Centre, Department of Biomedical Sciences, University of Sheffield, Western Bank, Sheffield S10 2TN, UK; 3 Departamento de Anatomía y Biología Celular, Facultad de Medicina, Universidad de Cantabria, Santander 39011, Spain

## Abstract

How the positional values along the proximo-distal axis (stylopod-zeugopod-autopod) of the limb are specified is intensely debated. Early work suggested that cells intrinsically change their proximo-distal positional values by measuring time. Recently, however, it is suggested that instructive extrinsic signals from the trunk and apical ectodermal ridge specify the stylopod and zeugopod/autopod, respectively. Here, we show that the zeugopod and autopod are specified by an intrinsic timing mechanism. By grafting green fluorescent protein-expressing cells from early to late chick wing buds, we demonstrate that distal mesenchyme cells intrinsically time *Hoxa13* expression, cell cycle parameters and the duration of the overlying apical ectodermal ridge. In addition, we reveal that cell affinities intrinsically change in the distal mesenchyme, which we suggest results in a gradient of positional values along the proximo-distal axis. We propose a complete model in which a switch from extrinsic signalling to intrinsic timing patterns the vertebrate limb.

## Abstract (web summary)

The specification of positional values along the proximo-distal axis (shoulder to digits) of the vertebrate limb is an unresolved issue. By using heterochronic transplants of distal mesenchyme, the authors show that the zeugopod and autopod (elbow to digits) are progressively specified in an intrinsically timed manner.

## Graphical abstract

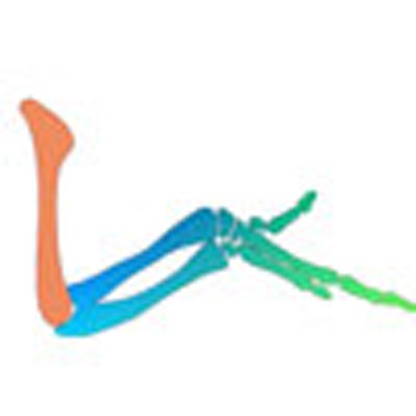

An enigmatic problem in developmental biology is how the positional values along the proximo-distal axis (that is, humerus to digits) of the vertebrate limb are specified. Although this has been a topic of intense investigation, a consensus model has not been reached. Currently, a variation on a two-signal model involving proximal signals from the trunk and distal signals from the apical ectodermal ridge (AER)—the thickened epithelium that rims the distal mesenchyme of the limb—is favoured. Mercader *et al.*^1^ proposed a model in which AER-derived fibroblast growth factors (FGFs) repress a proximal programme that specifies the stylopod (that is, humerus), leaving it unclear as to how the more distal zeugopod (that is, radius/ulna) and autopod (that is, wrist/digits) structures are specified. Instead, Mariani *et al.*^2^ suggested that instructive FGF signals specify presumptive autopod structures early, and that then intercalation between this domain and the presumptive stylopod results in the specification of the zeugopod. However, this sequence of proximo-distal specification has not been confirmed, as no molecular markers are known. Recently, work on the chick limb has shown that the stylopod is specified by diffusible signals, the identity of which is considered to be retinoic acid^3^,^4^, although this is controversial^5^. Therefore, how the zeugopod and autopod segments are specified remains contentious and some patterns of gene expression in the chick wing bud are not consistent with a signal-based mechanism. Thus, *Hairy2* (a gene involved in the somitogenesis clock) displays an oscillatory expression pattern^6^ and *Hoxa13* (an autopod marker), cannot be prematurely activated by AER signals that maintain its expression^7^,^8^. It has been proposed that *Hoxa13* expression is more consistent with the earlier progress zone model^9^ (for recent discussions see refs 10, 11). In the progress zone model, derived from embryological experiments on the chick wing bud, timing by an intrinsic clock operating in distal mesenchyme cells (the progress zone) is proposed to specify proximo-distal positional values^9^. An additional feature of this model is that extrinsic AER signals give distal mesenchyme cells the competence to measure time according to their own intrinsic clock.

To distinguish between intrinsic and signal-based timing mechanisms for specifying the zeugopod and autopod segments, we performed heterochronic transplants of small blocks of distal mesenchyme cells (150 μm) from young green fluorescent protein (GFP)-expressing^12^ donor chick wing buds underneath the intact AER of older wild-type host wing buds: a procedure that preserves the host limb bud architecture and incorporates the donor tissue within the signalling environment of the host distal mesenchyme. It is predicted that, if proximo-distal specification is intrinsically timed, then grafts of HH20 distal cells made to older (by 24 h) HH24 buds should behave according to the age of the donor wing bud; but if fate is controlled by extrinsic signalling, then the grafted cells should be re-specified and develop according to the host age. Using this technique, we reveal that distal cells develop according to their own age and that the positional values of the zeugopod and autopod are progressively specified in an intrinsically timed manner.

## Results

### The host environment appears to determine distal graft fate

**Figure 1:**
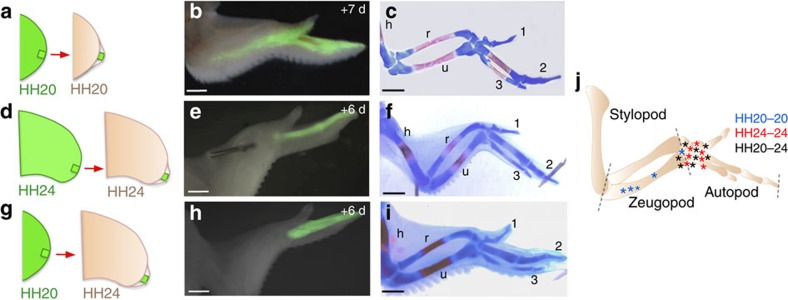
The environment appears to determine distal graft fate. Blocks (150 μm) of GFP-expressing (depicted as green in this and all schematic representations) HH20 chick wing distal mesenchyme denuded of ectoderm and grafted under the AER of wild-type HH20 buds (**a**) give rise to structures distal to the stylopod (*n*=5/5, **b**,**c**, blue asterisks—**j**). GFP-expressing HH24 distal mesenchyme tissue grafted beneath the AER of wild-type HH24 buds (**d**) give rise to structures distal to the zeugopod (*n*=7/7, **e**,**f**, red asterisks—**j**). GFP-expressing HH20 distal mesenchyme tissue grafted beneath the AER of wild-type HH24 buds (**g**) give rise to structures distal to the autopod (*n*=8/8, **h**,**i**, black asterisks—**j**). Each asterisk represents the proximal boundary of the grafted tissue for each experiment. Note, h-humerus; u-ulna; r-radius; 1, 2 and 3 are the digits in anterior to posterior sequence. Scale bars: 1 mm.

Earlier dye-based fate maps revealed that HH20 cells at the distal mesenchyme of the chick wing give rise to the zeugopod (radius/ulna), and at HH24, the autopod (wrist/digits)^13^,^14^. To see if our grafting technique could replicate these earlier fate maps, we performed homochronic grafts of 150 μm blocks of HH20 and HH24 GFP-expressing chick wing distal tissue under the AER of wild-type host buds (Fig. 1a,d). On days 11–12 of development, the GFP-labelled cells gave rise to the same structures along the proximo-distal axis as mesenchyme cells labelled at equivalent stages with dyes^13^,^14^—zeugopod and autopod with HH20 grafts (Fig. 1b,c—blue asterisks, j) and autopod only with HH24 grafts (Fig. 1e,f—red asterisks, j). These fate maps show that grafts of distal mesenchyme cells incorporate well and develop like host tissue. To understand if extrinsic signals or an intrinsic timing mechanism determines the proximo-distal positional values of grafted cells, and hence the structures that they contribute to, we made heterochronic grafts of blocks of HH20 distal tissue to HH24 buds (Fig. 1g). Analyses of the fate maps on days 11–12 revealed that these grafts gave rise to structures distal to the zeugopod (Fig. 1h,i—black asterisks, j) comparable with the presumptive fate of host HH24 distal cells and not donor HH20 cells (Fig. 1j). Therefore, this finding suggests that the signalling environment provided by the age of the host determines the positional value of grafted chick wing distal mesenchyme cells.

### *Hoxa13* is intrinsically timed in distal cells

**Figure 2:**
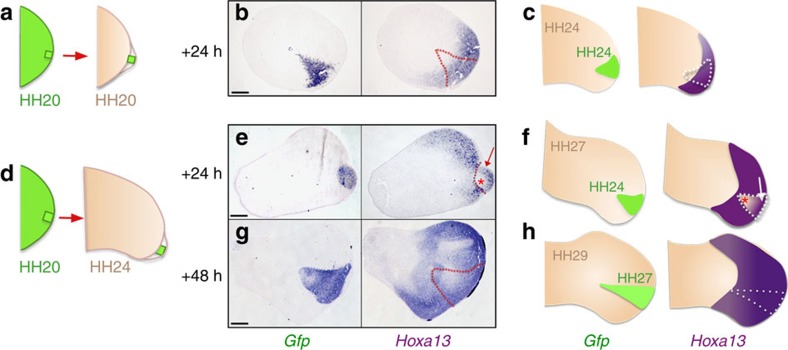
*Hoxa13* expression is intrinsically timed. In grafts of HH20 distal mesenchyme cells made to the same stage wing buds (**a**), the presence of the grafted tissue does not perturb the establishment of a normal domain of *Hoxa13* expression. Note that the graft cannot be distinguished by *Hoxa13* expression at 24 h. (**b**,**c**) *n*=2/2—note area of grafted tissue is shown by *Gfp* expression in a serial section (**b**) and dashed lines. In grafts of GFP-expressing HH20 distal mesenchyme tissue grafted beneath the AER of wild-type HH24 buds (**d**) *Hoxa13* is expressed in an intrinsically timed manner shown 24 h (**e**,**f**—*n*=3/3) and 48 h after grafting (**g**,**h**—*n*=3/3). Note that the arrows in (**e**,**f**) indicate *Hoxa13* expression in distal part of graft and asterisk absence in proximal part of the graft. Scale bars: 100 μm.

Previous evidence suggests that AER-derived FGFs in the chick wing bud are required to remove retinoic acid signals from the flank that inhibit the epigenetically timed programme of expression of the distal autopod marker, *Hoxa13* (refs 7, 8) (see normal expression pattern—Supplementary Fig. 1). Therefore, this is not consistent with the positional values of distal mesenchyme cells being determined by the age of extrinsic signalling environment (Fig. 1g–j). To address this discrepancy, we asked whether, in our heterochronic grafting assay, *Hoxa13* expression reflects the age of the donor distal mesenchyme or the host-signalling environment. In control homochronic grafts of HH20 distal mesenchyme cells made to HH20 buds (Fig. 2a), activation of *Hoxa13* expression occurred concomitantly in host and donor tissue and a normal pattern was observed after 24 h, showing that the grafting process does not affect the dynamics of *Hoxa13* transcription (Fig. 2b,c, note serial sections hybridized with *Hoxa13* and *Gfp* riboprobes to show the graft). However, in heterochronic HH20 grafts made to HH24 buds (Fig. 2d), *Hoxa13* expression in the graft was undetectable until around 24 h after grafting (Fig. 2e,f). In fact, transcripts were first detected in the distal part of the grafted tissue (arrows—Fig. 2e,f—then at HH24) and were absent in the proximal part (asterisks Fig. 2e,f), despite the entire graft being surrounded by *Hoxa13*-expressing host tissue (then at HH27—see also Supplementary Fig. 2). By 48 h, *Hoxa13* was expressed throughout the graft (then at HH27) and was indistinguishable from host tissue expression (then at HH29, Fig. 2g,h). Together, these data show that, despite the age of the extrinsic environment appearing to dictate the proximo-distal level that heterochronic grafts contribute to (Fig. 1), the activation of *Hoxa13* expression is timed on donor schedule. In addition, our results confirm that AER signals, either directly or indirectly, are required for *Hoxa13* induction, since grafts made to the presumptive zeugopod do not express *Hoxa13* (Supplementary Fig. 3). In addition, the rapid proximal spread of *Hoxa13* expression within the graft suggests that the establishment of the mature *Hoxa13* expression domain (Supplementary Fig. 1) is not just due to proliferation of a small founder population of sub-AER HH22 distal mesenchyme cells^15^, but also due to transcriptional initiation in progressively more proximal tissue. An alternative explanation for the distal restriction of *Hoxa13* expression in heterochronic grafts is that more proximal cells selectively die, but TUNEL analyses revealed no evidence of abnormal apoptosis (Supplementary Fig. 2).

### AER duration is timed by the distal mesenchyme

**Figure 3:**
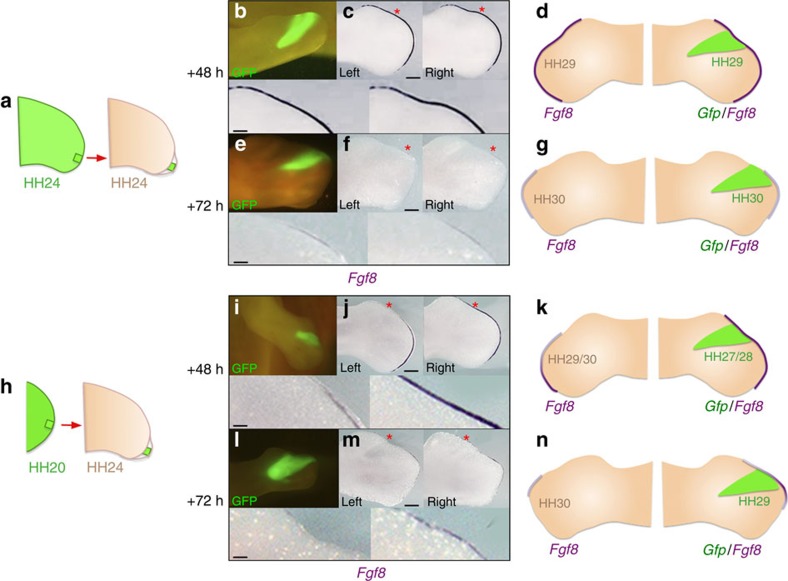
AER duration is locally controlled by the distal mesenchyme. In grafts of HH24 distal mesenchyme cells made to the same stage wing buds (**a**), the pattern of *Fgf8* expression in the AER is indistinguishable between left and right buds after 48 h (**b**–**d**, *n*=2/2) and 72 h (**e**–**g** *n*=2/2). Note area of grafted tissue is shown by GFP expression (**b**,**e**) and lower panels are higher magnifications of areas marked with asterisks (**c**,**f**). In grafts of GFP-expressing HH20 distal mesenchyme tissue grafted beneath the AER of wild-type HH24 buds (**h**) *Fgf8* is stronger in manipulated right buds compared with equivalent region of contralateral left buds after 48 h (**i**–**k**, *n*=2/2) and 72 h (**l**–**n**, *n*=2/2). Note area of grafted tissue is shown by GFP expression (**i**,**l**) and lower panels are higher magnifications of areas marked with asterisks (**j**,**m**). Left limb photos flipped horizontally for comparison in (**c**,**f**,**j**,**m**). Scale bars in (**c**,**f**,**j**,**m**)—100 μm and in their enlarged panels beneath—25 μm.

The AER is maintained by signalling from the underlying mesenchyme^16^,^17^ and is required for limb outgrowth until it regresses at around HH29/30 in the chick wing^18^. Therefore, since *Hoxa13* expression is intrinsically timed, we asked if the capacity to maintain the AER is also an intrinsic property related to the age of the distal mesenchyme, by examining *Fgf8* expression. During normal development, high-level *Fgf8* expression abruptly terminates at around stage HH29, first in the AER over the interdigits and then over the digits^19^. In control homochronic grafts of HH24 distal mesenchyme cells made to HH24 buds (Fig. 3a), after 48 h (HH29), *Fgf8* expression was indistinguishable between right-hand experimental and left-hand contralateral buds (asterisks, Fig. 3b–d). At 72 h (HH30), only residual *Fgf8* expression could be detected in left and right buds, showing that transcription was terminated at the same time (asterisks, Fig. 3e–g). Instead, in heterochronic grafts of HH20 distal tissue made to HH24 host buds (Fig. 3h), after 48 and 72 h, *Fgf8* was expressed in the AER overlying the grafted tissue at higher levels than in the equivalent AER region of the contralateral bud (asterisks, Fig. 3i–n). Therefore, the expression of *Fgf8* in the AER over the graft was extended for about 24 h longer than in the corresponding region of contralateral limb AER—equivalent to the difference in age between host and donor tissue. This result demonstrates that distal mesenchyme cells can locally maintain the AER in an intrinsically timed manner.

### Distal cell cycle parameters are intrinsically timed

**Figure 4:**
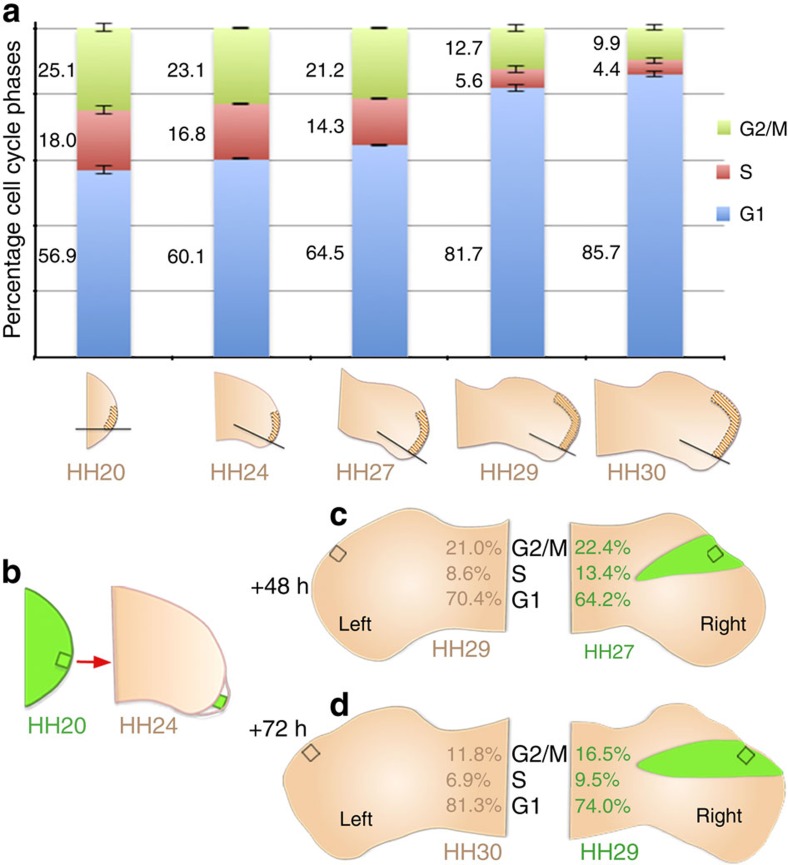
Cell cycle parameters are intrinsically timed. Cell cycle parameters of HH20 (three repeat experiments to account for variation in staging), HH24 (6), HH27 (6), HH29 (2) and HH30 (5) distal mesenchyme tissue—bars indicate standard error of the mean between the repeat experiments (**a**). In each experiment between 8,000 and 10,000 cells were counted from 10 to 12 pooled strips of distal tissue each obtained from separate buds. Blocks (150 μm) of GFP-expressing HH20 distal mesenchyme tissue grafted beneath the AER of wild-type HH24 buds (**b**), after 48 h (**c**), G1=64.2%, S=13.4%, G2/M=22.4% phase cells in grafted right wing distal mesenchyme blocks (150 μm) compared with G1=70.4%, S=8.6%, G2/M=21.0% phase cells in equal numbers of contralateral left wing bud distal mesenchyme blocks (one pool of *n*=10 cubes of tissue, each taken from separate left control and experimental right buds). After 72 h (**d**), G1=74.0%, S=9.5%, G2/M=16.5% of cells in grafted right wing distal mesenchyme blocks (150 μm) compared with G1=81.3%, S=6.9%, G2/M=11.8% of cells in equal numbers of contralateral left wing bud distal mesenchyme blocks (one pool of *n*=10 cubes of tissue in both cases). In both experiments, more than 10,000 cells were counted and there is a significant difference in G1, S and G2/M numbers between left and right distal mesenchyme (Pearson’s *χ*^2^ test—*P*<0.0001) consistent with graft behaving intrinsically.

We have previously shown that the intrinsic behaviour of Sonic hedgehog-producing polarizing region cells of the chick wing bud is linked with stage-specific cell cycle parameters^20^. Therefore, to test if adjacent non-polarizing distal mesenchyme cells have stage-specific cell cycle parameters, we undertook flow cytometric analyses at a range of stages between HH20 and HH30 (equivalent to the distal 150 μm tissue used in grafting experiments). We found that the proportion of chick wing distal mesenchyme cells in G1-phase increases from 56.9 to 64.5% between stages HH20 and HH27 (∼48 h), then abruptly to 85.7% by HH30 (∼96 h after HH20) indicating a loss of proliferative potential (Fig. 4a) that is comparable with the cells of the polarizing region^20^. Accordingly, the proportion of S-phase cells decreases from 18.0 to 4.4% and G2/M-phase cells decreases from 25.1 to 9.9% over the same time interval (Fig. 4a). To examine whether, as with the polarizing region, the cell cycle parameters of the adjacent distal mesenchyme are intrinsically controlled, we carried out flow cytometry on cells from grafts of HH20 distal tissue made under the AER of HH24 buds (Fig. 4b). By 48 (host at HH29) and 72 h (host at HH30), the proportion of G1-phase cells in the distal most 150 μm of the grafted tissue was significantly less (6.2 and 7.3% reduction, respectively) than in equivalent host tissue in contralateral buds (Fig. 4c,d, G1-phase percentages differ by <2% in left and right distal mesenchyme cells of normal buds and also those with homochronic grafts, see Supplementary Tables 1 and 2). In addition, the proportion of cells in S and G2/M phases in grafted tissue was significantly higher than in host tissue, consistent with increased proliferative potential (Fig. 4c,d). Indeed, the cell cycle phase values of grafted cells are similar to those expected for the stages of the younger donor embryos (HH27 and HH29—Fig. 4a). These data show that the cell cycle parameters of chick wing distal mesenchyme cells are intrinsically regulated.

### Distal positional values are intrinsically timed

Having demonstrated that grafts of HH20 distal mesenchyme cells made to HH24 buds intrinsically time *Hoxa13* expression, AER maintenance and cell proliferation, we investigated why their proximo-distal positional value appears to be influenced by the age of the host environment (Fig. 1h–j). Positional values are expressed as a gradient of cell adhesion along the proximo-distal axis of the limb^21^ and the differential adhesion between proximal and distal chick wing bud cells causes them to sort out in culture^22^,^23^. Therefore, we tested if the adhesive properties, and hence positional values of distal mesenchyme cells, are acquired in response to extrinsic signals or by an intrinsic timing mechanism *in vivo*. To achieve this, we disaggregated blocks of wild-type HH20 (zeugopod progenitor—Fig. 1j) and GFP-expressing HH24 (autopod progenitor—Fig. 1j) distal mesenchyme cells into single cells that were then re-aggregated and grafted to HH24 buds (see Materials and methods). Thus, if positional values are specified by host HH24 signals, then the grafted HH20 and HH24 cells are predicted to acquire equivalent adhesive properties and remain randomly dispersed in the grafts. Alternatively, if positional values are specified by an intrinsic timer, then grafted HH20 cells are not expected to be influenced by host HH24 signals, so should maintain adhesive properties reflective of their programmed fate, and thus sort out from grafted HH24 cells.

**Figure 5:**
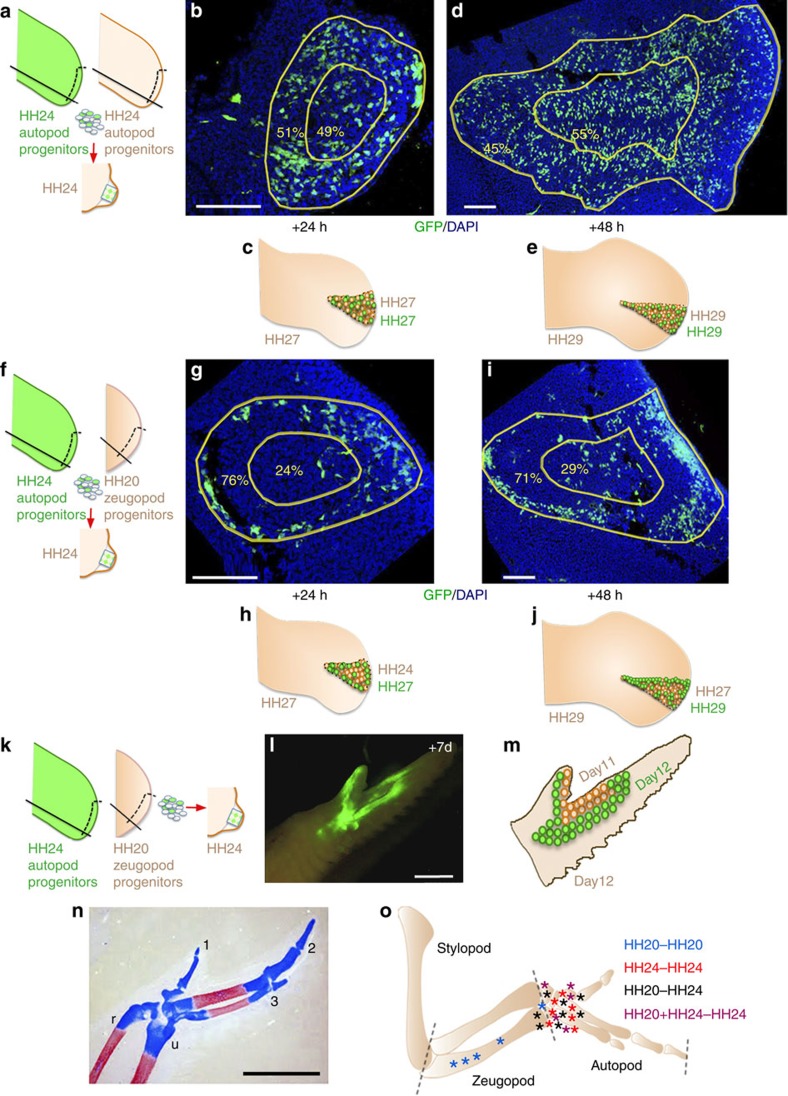
An intrinsic timer specifies distal positional values. Disaggregated GFP-expressing HH24 and wild-type HH24 autopod progenitor cells re-aggregated into pellets and grafted to HH24 buds (**a**) show an approximately equal distribution in the inner and outer regions of grafts after 24 (**b**,**c**, *n*=2/2) and 48 h (**d**,**e**, *n*=3/3, green shows GFP immunofluorescence and blue DAPI staining—the inner/outer regions defined as half the distance from the centre of the graft to the periphery as indicated by yellow rings). Disaggregated HH24 GFP-expressing autopod progenitor cells and wild-type HH20 zeugopod progenitor cells re-aggregated into pellets and grafted to HH24 buds (**f**) sort out after 24 h (**g**,**h**, *n*=3/3) and 48 h (**i**,**j**, *n*=3/3). Note the difference in cell distribution between the homochronic and heterochronic mixed grafts was very significantly different (Mann–Whitney–Wilcoxon *U*-test—*P*-value <0.0001). Autopod progenitor cells (then at HH27—**g** or HH29—**i**) predominantly localize to the periphery of the grafts (between the yellow rings) to aggregate with host cells of the same age and zeugopod progenitor cells (then at HH24—**g** or HH27—**i**) are confined to the centre of the grafts. Development of grafts comprising of HH24 GFP-expressing autopod progenitor cells and wild-type HH20 zeugopod progenitor cells made to HH24 buds (**k**). Autopod progenitor cells (then at day 12) localize to edge of grafted tissue to contact host cells of same age and zeugopod progenitor cells (then at day 11) are confined to the centre of the graft (**l**,**m** *n*=4/5). Such grafts contribute to the autopod (**n**,**o** –purple asterisks *n*=5/5; compare with HH20–20 grafts, blue asterisks; HH24-HH24 grafts, red asterisks and HH20–24 grafts black asterisks). Note in (**n**) u-ulna; r-radius; 1, 2 and 3 are the digits in anterior to posterior sequence). Scale bars—100 μm in (**b**,**d**,**g**,**i**; 1 mm in **l**,**n**).

In control grafts of GFP-expressing HH24 and wild-type HH24 distal mesenchyme cells made to HH24 buds (Fig. 5a), there appeared to be a random distribution of cells as assessed by GFP immunofluorescence (Fig. 5b–e—see Supplementary Fig. 4 for detection of *Gfp* mRNA). To quantitate this behaviour, we determined the distribution of GFP-expressing cells between the inner and outer regions of such grafts (inner/outer defined as half the distance from the centre of the graft to the periphery—see Materials and methods). At 24 h, the inner/outer distribution of grafted GFP-expressing HH24 cells was 49%/51% (Fig. 5b,c) and at 48 h, 55%/45% (Fig. 5d,e). On the other hand, grafts of GFP-expressing HH24 cells and wild-type HH20 cells made to HH24 buds (Fig. 5f) showed a non-random distribution (Fig. 5g–j, see Supplementary Fig. 4 for detection of *Gfp* mRNA). At 24 h, the inner/outer distribution of grafted GFP-expressing HH24 cells was 24%/76% (Fig. 5g,h) and at 48 h, 29%/71% (Fig. 5i,j). Therefore, this result shows that GFP-expressing HH24 distal mesenchyme cells had sorted out to the periphery of the graft, to associate with host tissue of the same age (then at HH27, Fig. 5g,h and HH29, Fig. 5i,j), In addition, this cell sorting confines wild-type HH20 distal mesenchyme cells (then at HH24, Fig. 5g,h and HH27, Fig. 5i,j) to the centre of the grafts (Supplementary Fig. 4). A similar pattern was still observed in 12-day-old wings thus showing that the distribution of cells caused by this earlier sorting event is maintained. Therefore, grafted GFP-expressing HH24 cells contact host cells of the same age (then both at day 12) and surround grafted HH20 wild-type cells (then at day 11, Fig. 5k–n). This result suggests that the inability of grafted HH20 zeugopod progenitor cells to contact host cells with equivalent positional values—that would have been displaced proximally at an earlier stage—entrains them into autopod structures and explains why grafts comprising HH20 cells made to HH24 buds are only able to contribute to the autopod (Fig. 5o—see Fig. 1h–j).

In summary, these data show that individual cells of different ages and proximo-distal fates sort out when grafted beneath the AER, thus indicating that the adhesive properties of cells, and hence positional values of the chick wing zeugopod and autopod, are specified by an intrinsic timer.

## Discussion

**Figure 6:**
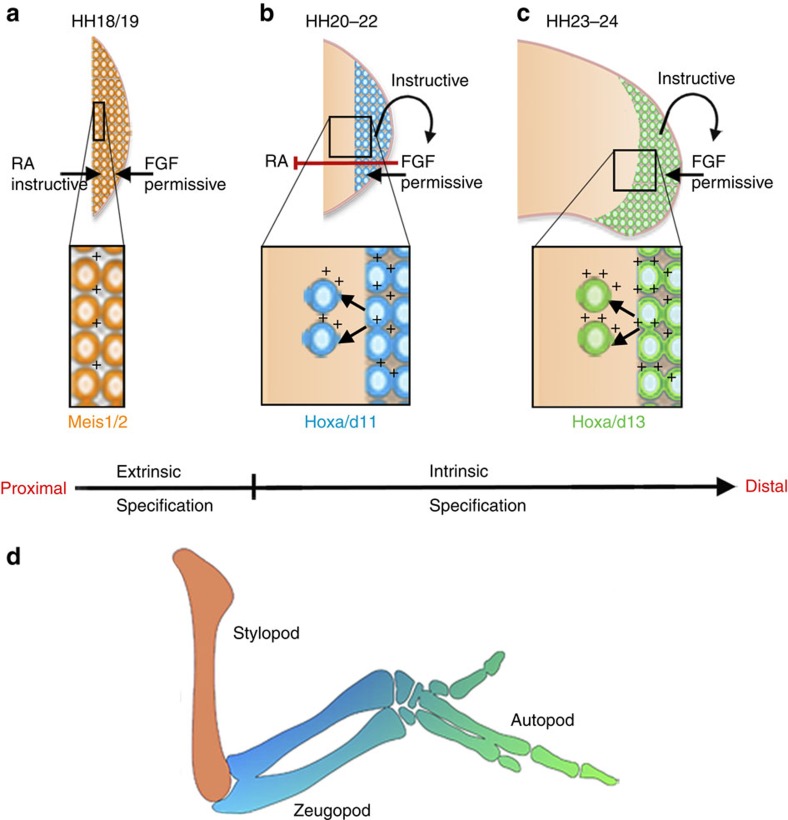
Model for chick wing proximo-distal patterning. At early limb initiation stages (HH18/19) trunk-derived retinoic acid (RA) specifies the positional value of the stylopod (humerus, **a**,**d**—orange) and then (HH20–22) intrinsic timing specifies the positional values of the zeugopod (forewing, **b**,**d** -blue) and later still (HH23–24) the autopod (wrist/digits, **c**,**d**—green). AER-derived FGFs are permissive factors that sustain growth of the limb bud and suppress the proximal programme including *Meis1/2* expression by inducing *Cyp26b1* that degrades retinoic acid (red line). Elimination of retinoic acid from the distal mesenchyme of the wing bud triggers the switch to intrinsic timing and mesenchyme cells express *5’Hoxa/d* genes and maintain AER-derived FGFs. Following the phase of proximal specification (**a**), cell adhesion properties and hence positional values intrinsically change over time (**b**,**c**, greater adhesion of cells shown by additional+symbols in lower insets) and this results in a spatial gradient of positional values along the proximo-distal axis as cells are displaced from the distal mesenchyme by an intrinsic programme of proliferation (arrows—lower insets **a**–**c**—graded blue/green on skeleton, **d**).

In this study, we set out to determine the contributions that extrinsic signalling and intrinsic timing mechanisms play in the specification of the distal structures of the limb. Our results provide evidence that an intrinsic timer, operating in distal chick wing mesenchyme cells, specifies the positional values of the zeugopod and autopod—consistent with the classical progress zone model^9^. Our findings allow us to present a complete model of proximo-distal limb patterning (Fig. 6a–d).

During limb initiation stages, trunk-derived retinoic acid specifies the stylopod^3^,^4^ (Fig. 6a—HH18/19 in the chick wing characterized by *Meis1/2* expression). Over time, AER-derived FGFs induce the retinoic acid-degrading enzyme *Cyp26b1* (ref. 24), and this, along with limb bud outgrowth, creates a retinoic acid-free distal mesenchyme domain (Fig. 6b,c). We propose that this event triggers an intrinsic timer in distal mesenchyme cells and the switch from proximal (stylopod) to distal (zeugopod/autopod) specification. Distal mesenchyme cells that activate this programme first transit through a phase of zeugopod specification (Fig. 6b—HH20–22, characterized by *Hoxa/d11* expression), and then a second phase of autopod specification (Fig. 6c—HH23–24, characterized by *Hoxa/d13* expression).

Importantly, we demonstrated that this intrinsic programme results in the acquisition of stage-specific cell adhesion properties, which are a read-out of proximo-distal positional values^21^,^22^,^23^. The fact that cells are being constantly displaced from the distal mesenchyme by an intrinsically timed programme of proliferation thus provides a robust mechanism by which a precise gradient of positional values can become established (Fig. 6d). Our findings therefore support the early idea that an intrinsic cell cycle clock^25^, sustained by AER signalling, is part of the timing mechanism that specifies the positional values of the zeugopod and autopod.

It has previously been shown that the switch from proximal to distal specification is associated with the epigenetic activation of the distal autopod marker, *Hoxa13* (ref. 8). We have extended these studies in showing that timing of *Hoxa13* expression is related to the age of the mesenchyme and is activated in a distal to proximal wave, which we suggest reflects the removal of inhibitory retinoic acid from the limb bud. Although it is unclear how the specification of positional values relates to the final limb anatomy, it is intriguing that *Hoxa13* regulates cell adhesion molecules such as *EphrinA7* (ref. 26). Therefore, given that the dosage of 5′ *Hoxa/d* gene products modulates the number of elements forming in the autopod by a Turing-type reaction-diffusion mechanism^27^, it is tempting to speculate that a link between this process of self-organization and cell adhesion exists.

Our finding that the intrinsic timing mechanism is not affected by the age of the AER supports the early proposal that AER signalling in the chick wing fulfils a permissive role in proximo-distal patterning (Fig. 6a–c)^28^, rather than the instructive role suggested by genetic experiments on the mouse limb^2^. Moreover, we showed that distal mesenchyme cells locally maintain the overlying AER for the appropriate duration to sustain outgrowth (Fig. 6b,c). It is possible that the AER and subjacent mesenchyme mutually maintain each other's maturation stage and that the AER reverts to a less mature stage upon our transplantation experiments. The possibility that permissive AER signals increase over time, which has never been demonstrated, cannot be excluded and requires further investigation.

A fundamental issue is whether extrinsic signals or an intrinsic timer re-specifies missing positional values during limb regeneration. It was recently demonstrated that amputated adult axolotl limbs regenerate missing structures in a proximal to distal sequence^29^, rather than by intercalation as previously suggested^30^. Thus, since it is difficult to conceive that signalling gradients can operate over the sheer size of the adult limb, an attractive alternative is that an intrinsic timer, recapitulating the embryonic one we have described here, governs limb regeneration—of at least the zeugopod and autopod segments. On a more general note, it is likely that the timers operating during proximo-distal limb patterning and somitogenesis share common components, such as the *Hairy2* gene, that has an oscillatory expression profile in both systems^6^,^31^. Moreover, an emerging theme is that both signal and time-based mechanisms operate together during embryogenesis^32^. Whether timing in other patterning systems is an intrinsic property remains largely undetermined.

## Methods

### Chick husbandry and tissue grafting

Wild-type and GFP-expressing fertilized Brown Leghorn chicken eggs were incubated, opened and staged according to Hamilton Hamburger^33^. For tissue grafts, wing buds of GFP-expressing HH20 or HH24 embryos were used, the posterior border containing the polarizing region was discarded and a stripe of 150 μm of distal sub-AER mesenchyme was dissected. The overlying ectoderm was dissected away after incubation in 0.25% trypsin at room temperature for 2 min and the mesenchyme stripe was then cut into two or three cuboidal pieces. These were then placed in slits made using a fine sharpened tungsten needle along the junction between the AER and subjacent mesenchyme in the mid-distal region of wing buds of normal host embryos at HH20 or HH24. Hosts were immediately returned to the incubator and harvested for analyses as desired. Before fixation, the development of the graft was examined and photographed under UVA light. For the grafts of re-aggregated distal tissue, stripes of distal tissue of GFP-expressing and wild-type wing buds were obtained following the above protocol. The tissue was disaggregated to single cells by gentle pipetting that were counted to make a ratio of 1 GFP-expressing to seven wild-type cells. Pilot experiments determined this ratio of labelled versus unlabelled cells was optimal for detection of labelled cells. The cells were then centrifuged at 2,500 r.p.m. for 8 min and resulting pellets were incubated for 20 min at 38 °C for consolidation, and then sectioned to generate blocks of 150 μm for tissue grafts.

### *In situ* hybridization

Digoxigenin-labelled antisense riboprobes were prepared for *Gfp*, *Fgf8* and *Hoxa13*. *Gfp* (*Aequorea victoria*) is the full-length open reading frame, cloned into the pGEM T easy vector (Promega) and transcribed with SP6 polymerase (Roche); *Fgf8* (*Gallus gallus*) is the full-length open reading frame cloned into pBlueScript and transcribed with T7 polymerase (Roche); *Hoxa13* (*Gallus gallus*) is a partial clone (80–290 of a 290 amino acid protein) in pBlueScript and transcribed with T3 polymerase (Roche). Briefly, for in situ hybridization in tissue sections, the samples were fixed overnight in 4% PFA, dehydrated, cleared, embedded in paraffin and sectioned at 7 μm. Consecutive sections were placed on separate slides to be analysed with different probes. The sections were de-paraffined, rehydrated, mildly digested with proteinase K (10 μg ml^−1^ for 10 min) and hybridized overnight. Sections were then washed with decreasing concentrations of SSC in 50% formamide (at 65 °C), and then blocked at room temperature with 10% sheep serum before overnight incubation at 4 °C in the standard anti-digoxigenin antibody conjugated to alkaline phosphatase (Roche,1:2,000). Finally, the staining reaction was carried out with NBT/BCIP and allowed to develop for the desired time.

### Immunofluorescence and quantification of cell distribution

Detection of GFP-expressing cells was performed by immunofluorescence on 7 μm paraffin sections. The sections were de-waxed, and blocked for 1–2 h with blocking buffer (1% bovine serum albumin, 1% goat serum in phosphate-buffered saline (PBS)). Cells were then incubated overnight at 4 °C with a rabbit polyclonal antibody for GFP (Life technologies) diluted 1:1,000 in washing buffer (blocking buffer diluted 1:10 in 1 × PBS), and immune complexes were detected with an Alexa-488 conjugated anti-rabbit antibody (Invitrogen) diluted 1:250 in washing buffer (in all cases, fluorescence was only observed in transgenic tissue expressing GFP and not in wild-type tissue). Finally, sections were counterstained with 0.5 mg ml^−1^ of DAPI, mounted in Vectashield and 1.5 μm thick Z-stacks (five optical sections per Z-stack) were acquired using a SP-5 laser-scan confocal microscope (Leica Microsystems) with a × 20, 0.7 NA objective, a 2 Airy pinhole and 400 Hz scanning speed. Cells were excited sequentially with 405 and 488 nm laser lines and fluorescence emission captured between 415 and 480 nm (DAPI) and 499 and 578 nm (GFP). Image analyses were performed with Image J: a maximum projection of the Z-stacks was generated after images were median-filtered to reduce noise. First, the perimeter of the graft was used to create an initial region of interest that was reduced by 50% using the transform tool in Adobe Photoshop to generate an inner region and outer region. Following this, the images were automatically thresholded and the GFP+ and DAPI+ area within the inner and outer regions of the graft were determined. The GFP+/ DAPI+ areas were calculated and compared between the two types of graft in both the outer and inner regions. To quantitate the immunofluorescence, three sections from each specimen were analysed. The proportion of the area occupied by the GFP-positive cells in the inner and outer regions of the grafts was determined. The calculation of the *P*-value was performed using three tests of the R software (that is, two *t*-tests with and without the homoscedasticity assumption and the nonparametric Mann–Whitney–Wilcoxon *U*-test).

### TUNEL-labelling

Detection of apoptosis was performed in sections of paraffin embedded tissue (7 μm) using terminal deoxynucleotidyl transferase mediated dUTP nick-end labelling (TUNEL) with the Apoptag Fluorescein Direct In Situ Apoptosis Detection Kit (Intergen) following the manufacturer’s instructions.

### Flow cytometry

Wild-type and GFP-expressing distal mesenchyme tissue was dissected in ice cold PBS under a LeicaMZ16F UV microscope using a fine surgical knife pooled from replicate experiments (between 10 and 12), and digested into single cell suspensions with trypsin (0.5%, Gibco) for 30 min at room temperature. Cells were washed in PBS (2 × ), fixed in 70% ethanol overnight, washed in PBS (2 × ) and re-suspended in PBS containing 0.1% Triton X-100, 50 μg ml^−1^ of propidium iodide and 50 μg ml^−1^ of RNase A (Sigma). Dissociated cells were left at room temperature for 20 min, aggregated cells were removed by filtration and the single cells then analysed for DNA content with a FACSCalibur flow cytometer and FlowJo software (Tree star Inc). Based on ploidy values cells were assigned in G1, S or G2/M phases and this was expressed as a percentage of the total cell number (approximately 10,000 in each case). To obtain stage-specific cell cycle profiles between three and six replicate experiments (pools of 10–12 blocks of tissue from separate embryos in each case) were performed and the standard error was calculated. Statistical significance of numbers of G1, S and G2/M phase cells between pools of left and right cubes of distal mesenchyme cells (10–12 in each cases) from the same embryos in homochronic, heterochronic grafting experiments—and also left and right controls was determined by Pearson’s *χ*^2^ tests to obtain two-tailed *P*-values (significantly different being a *P*-value of <0.05).

## Additional information

**How to cite this article:** Saiz-Lopez, P. *et al.* An intrinsic timer specifies distal structures of the vertebrate limb. *Nat. Commun.* 6:8108 doi: 10.1038/ncomms9108 (2015).

## Supplementary Material

Supplementary Information Supplementary Figures 1-4 and Supplementary Tables 1-2
