# Supplementary material for: An intrinsic timer specifies distal structures of the vertebrate limb
Source: Nat Commun. 2015 Sep 18;6:8108. doi: 10.1038/ncomms9108 (PMC4582416; doi:10.1038/ncomms9108)
Supplement: Supplementary Information — Supplementary Figures 1-4 and Supplementary Tables 1-2 [file ncomms9108-s1.pdf]

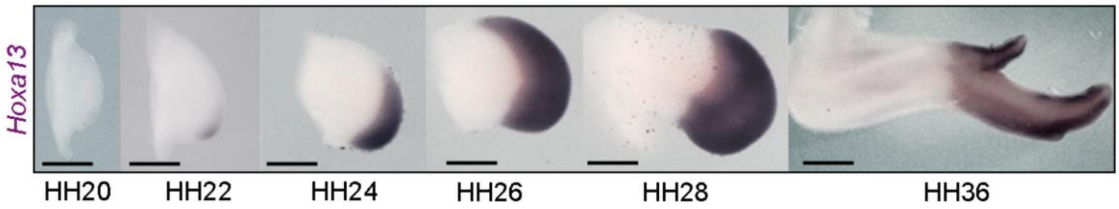

Supplementary Figure 1 - *Hoxa13* expression during chick wing development

Transcripts of *Hoxa13* are first detected in distal-posterior sub-AER mesenchyme cells at HH22 and are then detected in autopod cells throughout development. Scale bars - HH20, HH22, HH24, HH26 and HH28 – 500 µm: HH30 – 1mm.

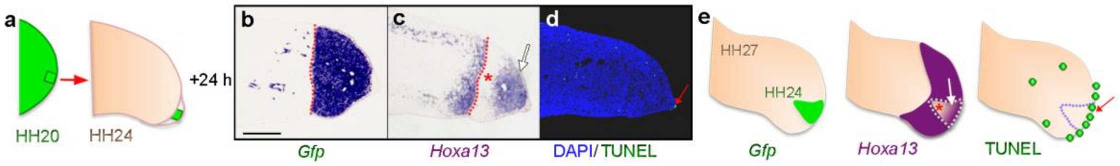

Supplementary Figure 2 - *Hoxa13* expression in grafts is not lost by apoptosis

In grafts of HH20 made to HH24 wing buds (**a**), after 24h, *Hoxa13* expression becomes expressed in the distal part of the graft (white arrows in **c**, **e**) and is absent in the proximal part (asterisk in **c**, **e**) despite the presence of more-proximal host expression**.** The area of graft is shown in a consecutive section hybridized with a *Gfp* riboprobe (**b)** and the proximal limit of graft is shown by dashed red line (**b**, **c**). The differential expression of *Hoxa13* in the graft is not due to loss of proximal graft tissue by apoptosis as shown by TUNEL labeling (**d**; blue is DAPI staining) in serial sections to those hybridized for *Gfp* and *Hoxa13*. Note, cell death is found in the AER as expected (red arrows, **d**, **e**). Schematics of the gene expression domains and cell death distribution are shown in **e**. Note, green spots indicate apoptotic cells and **b-d** are longitudinal sections. Scale bar – 100 µm.

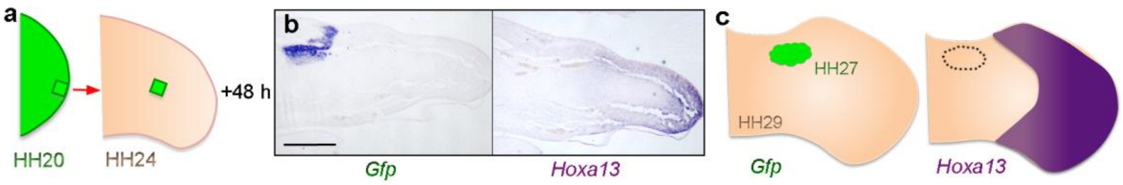

Supplementary Figure 3 – *Hoxa13* is not expressed in grafts made proximally

In grafts of HH20 distal tip cells made proximally to the presumptive zeugopod of HH24 buds (**a**) *Hoxa13* is undetectable after 48 h (**b, c**), although distal host expression is observed. Note area of grafted tissue is shown by *Gfp* expression (**b**, **c**). Scale bar – 100 µm.

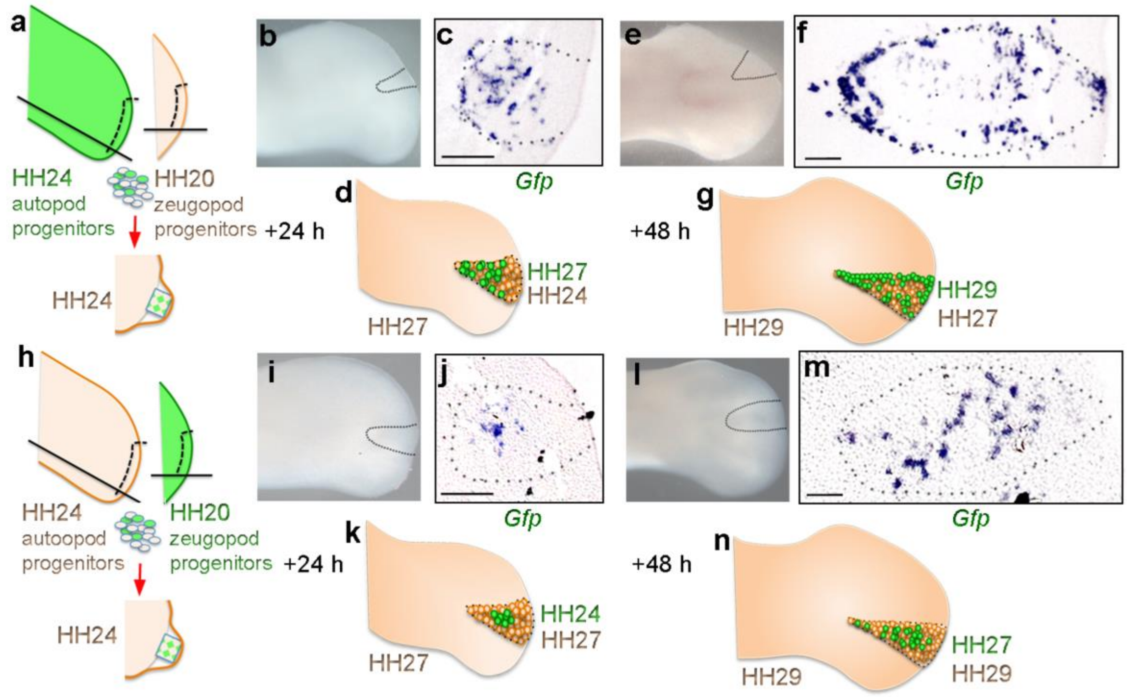

Supplementary Figure 4 - Sorting of autopod and zeugopod progenitor cells Disaggregated GFP-expressing HH24 autopod progenitor cells and wild type HH20 zeugopod progenitor cells re-aggregated into pellets and grafted to HH24 buds (**a**) sort out after 24 h (**b-d**) and 48 h (**e-g**). In both cases (*n*=2/2), RNA in situ hybridization for *Gfp*, shows that HH24 cells (then at HH27 or HH29) predominantly localise to the periphery of the grafts (dashed lines) to contact host tissue of the same age, leaving wild type HH20 cells confined to centre of the grafts (then at HH24 or HH27). Disaggregated GFP-expressing HH20 zeugopod progenitor cells and wild type HH24 autopod progenitor cells re-aggregated into pellets and grafted to HH24 buds (**h**) sort out after 24 h (**i-k**) and 48 h (**l-n**). In both cases (*n*=2/2), HH20 cells (then at HH24 or HH27) are confined to the centre of the grafts, surrounded by wild type HH24 cells contacting host tissue of same age ((HH27 or HH29). Scale bars – 100 µm.

Supplementary Table 1 - Cell cycle profiles of left and right distal tips

Stage	G1	S	G2/M	Stage	G1	S	G2/M
HH24 (R)	58.7	15.7	25.6	HH24 (L)	58.2	16.3	25.5
HH27 (R)	64.5	12.1	23.4	HH27 (L)	63.7	12.7	23.6

At least 10,000 cells were counted from one pool of 10 distal mesenchyme blocks in each case. Pearson's χ^2 test on total cell numbers reveals no significant difference ($p > 0.05$) in percentages of cells in G1, S and G2/M phase in left (L) and right (R) buds.

Supplementary Table 2 - Cell cycle profiles of homochronic distal tip grafts

Graft	Right stage	G1	S	G2/M	Left stage	G1	S	G2/M
HH20-20	HH24	58.6	19.6	21.8	HH24	59.7	18.9	21.4
HH24-24	HH27	63.2	14.7	22.1	HH27	64.3	13.5	22.2

At least 10,000 cells were counted from one pool of 10 distal mesenchyme grafts and equivalent contralateral tissue in each case. Pearson's χ^2 test on total cell numbers reveals no significant difference ($p > 0.05$) in percentages of cells in G1, S and G2/M phase in contralateral (L) and grafted (R) buds.
